# The co-occurrence of myocardial dysfunction and peripheral insensate neuropathy in a streptozotocin-induced rat model of diabetes

**DOI:** 10.1186/1475-2840-13-11

**Published:** 2014-01-11

**Authors:** Maria N Marangoni, Scott T Brady, Shamim A Chowdhury, Mariann R Piano

**Affiliations:** 1 Department of Anatomy and Cell Biology, University of Illinois at Chicago, 808 S. Wood St. Rm 578 (M/C 512), Chicago, IL 60612, USA; 2 Department of Physiology and Biophysics, University of Illinois at Chicago, 835 S. Wolcott Ave (M/C 901), Chicago, IL 60612, USA; 3 Department of Biobehavioral Health Science, University of Illinois at Chicago, 845 South Damen Avenue, Room 706 (M/C 802), Chicago, IL 60612-7350, USA

**Keywords:** Insensate neuropathy, Systolic dysfunction, Diastolic dysfunction, Norepinephrine, Diabetic cardiomyopathy

## Abstract

**Background:**

Cardiomyopathy and distal symmetrical polyneuropathy (DSPN), including sensory and autonomic dysfunction, often co-occur in diabetic mellitus (DM) patients. However, the temporal relationship and progression between these two complications has not been investigated. Using a streptozotocin DM animal model that develops insensate neuropathy, our aim was to examine in parallel the development of DSPN and DM-associated changes in cardiac structure and function as well as potential mechanisms, such as autonomic dysfunction, evaluated by changes in urinary and myocardial norepinephrine content and myocardial neuronal markers.

**Methods:**

Sensory neuropathy was measured by behavioral tests using Von Frey filaments and Hargreaves methods. Echocardiography was used to evaluate myocardial structure and function. Autonomic function was evaluated by measuring urinary and myocardial norepinephrine (NE) levels by enzyme-linked immunosorbent assay and high-performance liquid chromatography/mass spectrometry. Quantitative immunohistochemistry was used to measure the myocardial neuronal markers, calcitonin gene-related peptide (CGRP) and general neuronal protein gene product 9.5 (PGP 9.5).

**Results:**

The DM group developed tactile and thermal insensate neuropathy 4–5 weeks after DM onset. Cardiovascular changes were found between 4 and 12 weeks after DM onset and included bradycardia, diastolic and systolic dysfunction and cardiac dilation. There was a 2.5-fold reduction in myocardial NE levels and a 5-fold increase in urinary NE levels in the DM group. Finally, there was a 2.3-fold increase in myocardial CGRP levels in the DM group and no change in PGP9.5 levels.

**Conclusions:**

Cardiovascular structural and functional changes developed early in the course of DM and in combination with insensate neuropathy. In parallel, signs of cardiac autonomic dysfunction were also found and included decreased myocardial NE levels and altered CGRP levels. These results may indicate the need for early cardiovascular evaluation in DM patients with insensate neuropathy.

## Background

Diabetes mellitus (DM) is associated with many disabling complications, including nephropathy, retinopathy, polyneuropathy, and cardiomyopathy. Among these complications, polyneuropathy such as distal symmetrical polyneuropathy (DSPN) and other peripheral neuropathies are the most common long-term complication of DM affecting >50% of patients. Cardiovascular disease, however, is the leading cause of death. Adults with DM have cardiovascular disease death rates that are 2–3 times higher than adults without DM [1]. Cardiovascular complications can include coronary artery disease and diabetic cardiomyopathy. Diabetic cardiomyopathy occurs independently of underlying coronary artery disease or hypertension and is characterized by a dilated ventricular phenotype, as well as diastolic and systolic dysfunction [2].

Data from population-based studies support that DM complications often cluster and coexist. For example, Chung et al. reported that complications such as retinopathy and nephropathy occurred more often in type 2 DM patients with DSPN, a sensory neuropathy [3]. There is also evidence that co-occurrence of DSPN and cardiac autonomic neuropathy is associated with changes in ventricular structure and function [4,5]. Others have found that cardiac autonomic neuropathy is an independent predictor of cardiovascular disease mortality [6]. Despite the frequent co-occurrence of DSPN and myocardial dysfunction, the temporal relationship of the onset and progression of these complications has not been fully investigated. Insensate neuropathy is a pathogenic hallmark of DSPN and contributes to the high prevalence of unawareness or the asymptomatic nature of DSPN. Diabetes duration and blood glucose control/variability correlate with the development of DSPN; however, these mechanisms do not fully account for the onset of DM complications, such as DSPN, suggesting the involvement of other factors, such as loss of insulin signaling [7]. Diabetic cardiomyopathy also involves impairment of myocardial autonomic neurons and direct neuronal damage to afferent and efferent fibers, leading to cardiac autonomic dysregulation and silent myocardial infarction [8]. Several studies have evaluated myocardial norepinephrine (NE) levels and myocardial sympathetic and parasympathetic density separately, but no study has measured these variables together in the same animal model.

Therefore, using a non-insulin-dependent streptozotocin (STZ) DM animal model that develops insensate neuropathy, the purpose of this investigation was to examine in parallel the development of DSPN and DM-associated changes in cardiac structure and function, as well as potential mechanisms, such as autonomic dysfunction, evaluated by changes in urinary and myocardial NE content and myocardial neuronal markers. Myocardial neuronal markers evaluated included, calcitonin gene-related peptide (CGRP) a marker for sensory fibers, and PGP9.5, as general neuronal marker. In our STZ-DM animal model, we found early development of cardiovascular functional and structural changes in the presence of insensate neuropathy with signs of autonomic deficits.

## Methods

### Animals and induction of diabetes mellitus

A total of 38 adult male Fischer 344 rats (300 g, 16 week-old, Charles River, MA) were studied. Diabetes mellitus was induced with a single tail vein injection of STZ (Sigma-Aldrich Chemicals, St. Louis, MO, 35 mg/kg; Week 0, baseline). The control (CON) group received an equal volume of vehicle (0.1 mol/l citrate buffer, pH 4.5). Both groups received standard rat chow and water ad libitum. Animals were weighed at weekly intervals, and blood glucose levels were measured using the Freestyle glucose monitoring system (Therasense®, Alameda, CA). One week following STZ injection, all animals developed hyperglycemia with glucose values exceeding 300 mg/dL (STZ-DM group). Diabetic animals were monitored for polydipsia, polyuria, and weight loss. A onetime high dose of STZ in adult animals kills a proportion of pancreatic beta cells rendering the animal hypoinsulinemic and hyperglycemic but with sufficient residual insulin for short-term survival. All procedures were performed in accordance with the Guide for the Care and Use of Laboratory Animals published by the U.S. National Institutes of Health (National Institutes of Health, Publication No. 85–23, revised 1996) and approved by the University of Illinois at Chicago Institutional Animal Care and Use Committee.

### Behavioral testing

Development of sensory neuropathy was measured by two methods in CON (n = 12) and STZ-DM animals (n = 13). Animals were tested for behavioral responses to noxious thermal stimuli using the Hargreaves method [9] and to non-noxious sensory stimuli using flexible Von Frey filaments [10]. Both tests were performed before injections and weekly thereafter through Week 8. Animals were placed in individual cages, and behavioral accommodation was allowed until there was cessation of cage exploration and major grooming activities. Stimuli were presented to mid-plantar sections of both hind paws. Briefly, for Von Frey methods a floorless fiberglass box (18 × 8 ×8 cm) was placed on top of an elevated mesh floor for paw access. Von Frey threshold was calculated using the up-and-down method of Dixon [11]. For the Hargreaves method, rats were placed under an inverted clear fiberglass box (18 × 8 × 8 cm) on top of an elevated glass floor and allowed to acclimate. The radiant heat source (Ugo Basile Biological Research Apparatus Model 737150, Comerio, Italy) was positioned under the glass floor directly beneath the hind paw, and the thermal latency was measured in seconds.

### Echocardiography

All echocardiograms were performed as previously described by our laboratory and according to the American Society of Echocardiography guidelines [12]. Echocardiograms were performed in CON (n = 6) and STZ-DM animals (n = 7) at 4, 8, and 12 weeks by the same experienced sonographer using the Sequoia C256 Echocardiography System (Acuson Corporation, Mountain View, CA) and a 15.0 MHz transducer. Before the procedure, animals were anesthetized with an initial dose of methoxyflurane, and sedation was maintained thereafter via intubation with 1% isoflurane, using a Harvard small-animal ventilator (respiratory rate 80 breaths per minute, respiratory volume 2.5 ml). Body temperature was maintained at 37°C using a warming plate perfused with a water circulating bath. The transducer was placed on the left thorax, and M-mode and 2-dimensional echocardiography images were obtained in the papillary muscle level.

The measurements listed were obtained after a well-defined, continuous interface of the anterior and posterior walls was visualized. All parameters were measured with electronic calipers, and mean calculations were obtained from three or more consecutive cardiac cycles. After good visualization, left ventricular end-diastolic dimension (LVDD), left ventricular end-systolic dimension (LVSD) and interventricular septal thickness in diastole (IVSD) and in systole (IVSS) were measured by the leading edge method [13]. The former two parameters reflect intraventricular volume and therefore dilation of the left ventricle and IVSD and IVSS, reflect the wall thickness of the septal wall during diastole and systole. In all animals, three to four beats were recorded using the same transducer position. Fractional shortening (FS) and relative wall thickness (RWT) were calculated as previously described [12]. LV mass was calculated using the following equation: LV mass = 1.04 × [(LVDD + posterior wall thickness + IVSD)^3^ - LVDD^3^]. Using pulse-wave Doppler echocardiography, we also measured signals from the left ventricular inflow and outflow track and obtained indices of diastolic function. These included isovolumic relaxation time (IVRT), which reflects the time between the closure of the aortic valve and opening of the mitral value, deceleration time (DecT), and E/A ratio, in which the E wave represents early rapid filling of the ventricle and the A wave represents late filling of the ventricle. Mean values were used for statistical analysis.

### Urine and myocardial norepinephrine levels

At 8 weeks, in a subgroup of CON (n = 5) and STZ-DM (n = 6), we measured urinary and myocardial NE levels. Twenty-four hour urine samples were collected for the measurement of NE and creatinine [12]. The total 24 h volume was measured, and 1-ml aliquots (containing 6 mol/l hydrochloride) were stored at -20°C until assay. Norepinephrine was measured by enzyme-linked immunosorbent assay (Rocky Mountain Diagnostics, Colorado Springs, CO). All samples were run in duplicate and normalized to urinary creatinine to correct for urine volume [14]. Norepinephrine levels were expressed as mmol/l 24 h NE/creatinine.

Following the 24 hour urine collection, animals were anesthetized with CO_2_ and then euthanized by cervical dislocation. Hearts were excised, rinsed in phosphate buffered solution, weighed and snap frozen in liquid nitrogen and stored at -70°C. Catecholamine levels in biological samples can change over time due to auto-oxidation, exposure to light, air oxidation and/or exposure to high pH and temperature [15]. Therefore as noted below certain procedures/techniques were used when handling, storing and preparing samples to avoid degradation of NE. Frozen myocardial tissue samples were pulverized in liquid nitrogen and homogenized in glass homogenizers to minimize oxidation from metal homogenizers on ice in buffer (2 ml/g tissue) containing 0.1 N perchloric acid and 0.02 mmol/l EDTA. To control for oxidation of NE two homogenization buffers were tested, with and without sodium metabisulfite (see Additional file 1). Sodium metabisulfite, a commonly used antioxidant, quenched the NE signal and therefore was not used for the homogenization of samples. Homogenates were centrifuged (15,000 g, 20 min, 4°C), and the resulting supernatants were used for NE extraction and aliquots stored at -80°C. As previously described, samples were analyzed using a high-performance liquid chromatograph (Agilent 1200 series high performance liquid chromatography system, Santa Clara, CA) and Agilent Zorbax R_x_-C_8_ (150 × 2.1 mm I.D., 5 μm) column, along with a pre-column filter (used in the isocratic, reverse-phase, ion-paring mode) coupled with tandem mass spectrometry detection [16]. The mobile phase consisted of water–methanol-heptafluorobutyric acid (85/15/.13, V/V/V), with a flow rate of 0.2 ml min^-1^. The entire chromatographic effluent (10 μl) was passed through the mass spectrometer interface for subsequent detection. Under these conditions, NE retention time was about 4.4 min resulting in a total time (injection-to-injection) of 6 min. Deuterium-NE (d-NE) (Fisher #NC9750934, Pittsburgh, PA) was used as an internal standard. NE/d-NE area ratios were determined for the SRM chromatographic peaks using Agilent’s MassHunter software. Calibration curves were generated by plotting peak area ratios (NE/d-NE) obtained from working standard versus NE concentrations and fitting these data to a weighted (1/x) linear regression curve.

### Evaluation of neuronal markers

The myocardial neuronal markers calcitonin gene-related peptide (CGRP) and PGP9.5 were also evaluated at 8 weeks in a separate subgroup of CON (n = 3) and STZ-DM (n = 4) animals. After euthanasia as described above, hearts were removed and pulverized with a ceramic grinder under liquid nitrogen and homogenized on ice in cold buffer (10 ml/g tissue) containing: 50 mmol/l 4-(2-hydroxyethyl)-1-piperazineethanesulfonic acid buffer (pH 7.4), 5 mmol/l ethylenediaminetetraacetic acid, 150 mmol/l NaCl, 1% Triton X-100, 1X phosphatase inhibitor cocktail set II (Calbiochem #524625, Billerica, MA), 1X protease inhibitor cocktail (Sigma #P8340, St. Louis, MO), and 50 nmol/l okadaic acid. Homogenates were centrifuged at 14,000 rpm for 15 min at 4°C, and supernatants were used for blots. A small aliquot was reserved for protein quantification by bicinchoninic acid (Pierce #23225, Thermo Scientific, Hanover Park, IL). Supernatants with sample buffer were loaded onto a 4-12% Bis/Tris gradient gel (Invitrogen, Grand Island, NY) and separated under reducing conditions. Samples were incubated overnight at 4°C with primary antibodies against calcitonin gene-related peptide (CGRP, Millipore AB15360, Billerica, MA, 1:5,000), protein gene product 9.5 (PGP 9.5, Millipore AB1761, Billerica, MA, 1:2,000) and glyceraldehyde-3-phosphate dehydrogenase (GAPDH, Abcam 341048, Cambridge, MA, 1:5,000). Corresponding secondary antibodies conjugated with horseradish peroxidase enzyme goat anti-rabbit (Jackson ImmunoResearch, West Grove, PA, #111-035-045) and goat anti-mouse (Jackson ImmunoResearch, West Grove, PA, #115-035-044) were added to samples, and membranes were washed of unbound secondary antibodies, followed by incubation with peroxidase substrate for enhanced chemiluminescence and detection by film processor. Using Image J software (National Institutes of Health), optical densitometry was calculated and normalized to GAPDH.

### Statistical analyses

All data are expressed as mean ± SEM. Normally distributed data (body weight, glucose measurements, Hargreaves, and echocardiograms) were compared using two-way repeated measures analysis of variance (RM-ANOVA), followed by the Holm-Sidak method for multiple comparison. Student's *t* test was used for two-group parametric comparisons (urinary and myocardial NE, Immunoblots). The Mann–Whitney test was used to compare non-parametric data at each interval (von Frey filaments). A *p* value less than 0.05 was considered significant. Statistical analyses were performed using Sigmaplot (Systat Software, San Jose, CA).

## Results

### Diabetic animal model

Glucose levels measured at 4, 8, and 12 weeks were significantly greater in the STZ-DM animals compared to CON rats (Table 1). After DM induction and at all time points, body weight (BW) in STZ-DM animals was significantly lower than in CON animals (Table 1). STZ-DM animals exhibited polydipsia, polyuria, diarrhea, and weight loss. No STZ-DM animals required insulin administration and no animals were euthanized for animal welfare issues.

**Table 1:** Body weight and glucose in CON and STZ-treated F344 rats

**Groups**	**4 weeks**	**8 weeks**	**12 weeks**
	**Body weight (g)**		
CON (n = 6)	340 ± 5	378 ± 7	396 ± 10
STZ-DM (n = 7)	255 ± 9^a^	256 ± 13^a^	239 ± 13^a^
	**Glucose (mg/dL)**		
CON (n = 6)	87 ± 7	68 ± 4	76 ± 4
STZ-DM (n = 7)	370 ± 22^a^	373 ± 28^a^	398 ± 30^a^

Data are expressed as mean ± SEM. CON: control; STZ-DM: streptozotocin-induced diabetes mellitus. Two-way RM ANOVA, Holm-Sidak method. ^a^*p* < 0.001 STZ-DM vs CON.

### Evaluation of DSPN

**Figure 1:**
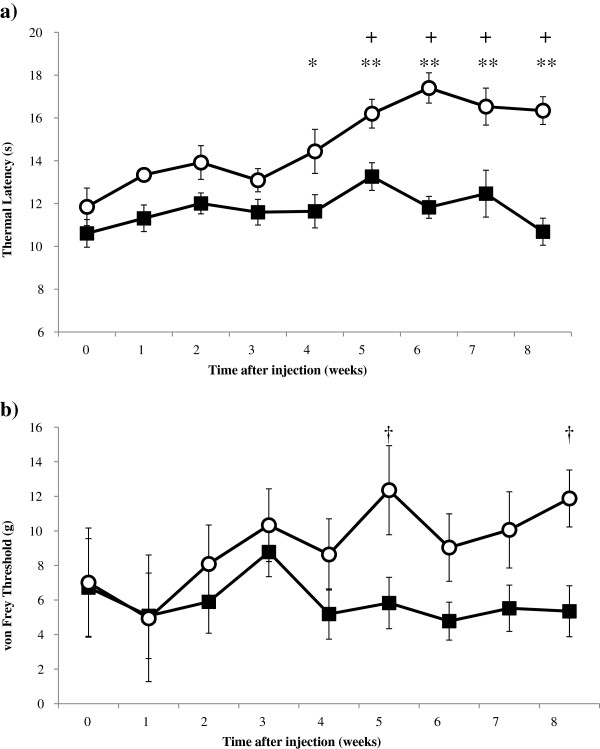
**Behavioral responses of rats to mechanical and thermal stimulation of hind paws.** Data are mean ± SEM. CON: control (black squares, *n* = 13); STZ-DM: streptozotocin-induced diabetes mellitus (white circles, *n* = 12). **(a)** Thermal latencies measured by Hargreaves method in CON and STZ-DM groups. Two-way RM ANOVA CON vs. STZ-DM: *p < 0.05; **p < 0.001; STZ-DM 5–8 weeks vs. Week 0 + *p* < 0.05. **(b)** Von Frey threshold was measured using up-and-down method in CON and STZ-DM groups. Mann–Whitney test CON vs STZ-DM. †*p* < 0.05.

Within the CON group, no changes in thermal latency or Von Frey threshold values were found over time (Figure 1, panel a and b, respectively). Within the STZ-DM group, compared to baseline (Week 0), significant increases in thermal latency were found at 5 through 8 weeks (Figure 1, panel a). Compared to the CON group, significant increases in thermal latency were found in the STZ-DM group at 4–8 weeks (Figure 1, panel a). Within the STZ-DM group, compared to baseline, no differences were found in Von Frey threshold over time. Compared to the CON group, significant increases were found in Von Frey threshold in the STZ-DM group at 5 and 8 weeks (Figure 1, panel b).

### Echocardiography

Within the CON group, no changes in echocardiography parameters were found over time (Table 2). At 8 and 12 weeks, heart rate was significantly lower in the STZ-DM group compared to the CON group (Table 2). At all time points, LVDD/BW and LVSD/BW were significantly greater in the STZ-DM group compared to the CON group (Table 2). Except for a significant increase in IVSS/BW in the STZ-DM group compared to the CON group at 4 weeks (*p* < 0.05), no differences in IVSD/BW or IVSS/BW were found between groups at other time points. At 4 weeks, left ventricular mass/BW was significantly lower in the STZ-DM group compared to the CON; while at 8 weeks, in the STZ-DM group, left ventricular mass/BW was significantly greater than that of the CON group, and no differences were found between groups at 12 weeks (Table 2). At all time points, the STZ-DM group compared to the CON group showed significantly lower RWT/BW; significantly lower fractional shortening (%); significantly greater IVRT; and significantly lower E/A ratio. No changes were found at any time point in deceleration time of the myocardium between groups.

**Table 2:** Echocardiography parameters in CON and STZ-DM rats

	**4 weeks**		**8 weeks**		**12 weeks**	
**Groups**	**CON**	**STZ-DM**	**CON**	**STZ-DM**	**CON**	**STZ-DM**
	**(n = 6)**	**(n = 7)**	**(n = 6)**	**(n = 7)**	**(n = 6)**	**(n = 7)**
HR (bpm)	330.7 ± 13.6	314.4 ± 26.4	349.7 ± 8.5	297.9 ± 8^a^	328.3 ± 18.7	277.7 ± 9.3^a^
LVDD/BW (mm/kg)	21.90 ± 0.4	30.90 ± 1.1^b^	19.70 ± 0.4	32.20 ± 1.5^b^	19.91 ± 0.63	35.25 ± 1.9^b^
LVSD/BW (mm/kg)	12.40 ± 0.5	19.50 ± 1.3^b^	11.20 ± 0.3	21.20 ± 1.3^b^	12.21 ± 0.39	25.64 ± 1.5^b^
IVSD/BW (mm/kg)	2.68 ± 0.13	3.08 ± 0.21	2.63 ± 0.14	2.99 ± 0.15	2.24 ± 0.11	2.50 ± 0.21
IVSS/BW (mm/kg)	5.35 ± 0.32	5.98 ± 0.47^a^	5.19 ± 0.28	5.55 ± 0.34	4.24 ± 0.25	4.47 ± 0.32
LVmass/BW (g/kg)	1.21 ± 0.03	0.80 ± 0.06^b^	1.20 ± 0.07	1.45 ± 0.08^a^	1.12 ± 0.06	1.25 ± 0.1
RWT/BW (mm/kg)	0.44 ± 0.02	0.37 ± 0.01^a^	0.43 ± 0.02	0.33 ± 0.01^b^	0.39 ± 0.02	0.27 ± 0.01^b^
FS (%)	43.7 ± 2.2	37.1 ± 2.4^a^	43.2 ± 1.4	32.6 ± 1.3^a^	38.8 ± 1.1	27.4 ± 0.7^a^
E/A ratio	1.98 ± 0.07	1.50 ± 0.05^a^	1.98 ± 0.13	1.55 ± 0.12^a^	1.83 ± 0.15	1.35 ± 0.02^a^
IVRT (msec)	26.3 ± 2	34.4 ± 3^a^	25.3 ± 1.7	32.9 ± 2.8^a^	27.7 ± 1.8	38.1 ± 2.9^a^
DecT (msec)	45.7 ± 0.8	41.6 ± 2.6	41.2 ± 2.9	47.9 ± 3.2	44.2 ± 2.8	43.3 ± 2.5

Data are expressed as mean ± SEM; CON: control; STZ-DM: streptozotocin-induced diabetes mellitus; HR: heart rate; BW: body weight; LVDD: left ventricular end-diastolic dimension; LVSD: left ventricular end-systolic dimension; IVSD: interventricular septum in diastole, IVSS: interventricular septum in systole; LV: left ventricle; RWT: relative wall thickness; FS: fractional shortening; E/A: E wave to A wave ratio; IVRT: isovolumic relaxation time; DecT: deceleration time. Two-way RM ANOVA Holm-Sidak test. ^a^*p* < 0.05 CON vs STZ-DM; ^b^*p* < 0.001 CON vs STZ-DM.

**Figure 2:**
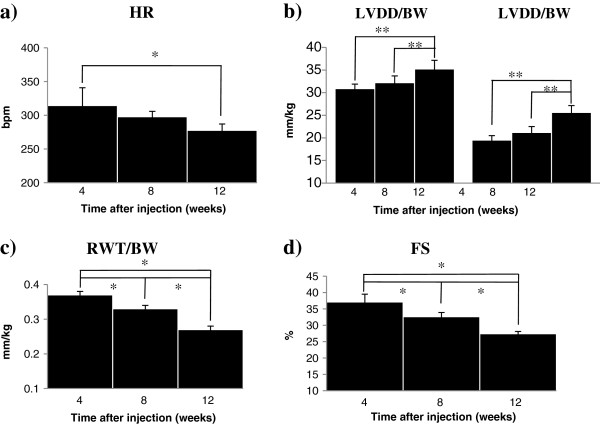
**Time course of changes in echo-derived parameters in STZ-DM animals. (a)** Heart rate (HR). Holm-Sidak 4 weeks vs 12 weeks. *p < 0.05. **(b)** Left ventricular end-diastolic dimension (LVDD/BW) and left ventricular end-systolic dimension (LVSD/BW), Holm-Sidak 4 weeks vs. 12 weeks and 8 weeks vs 12 weeks. **p < 0.001. **(c)** Relative wall thickness (RWT/BW). Holm-Sidak 4 weeks vs 12 weeks, 4 weeks vs 8 weeks, and 8 weeks vs 12 weeks. *p < 0.05. **(d)** Fractional shortening % (FS). Holm-Sidak 4 weeks vs 12 weeks, 4 weeks vs 8 weeks, and 8 weeks vs. 12 weeks. *p < 0.001. BW: body weight.

Over time in the STZ-DM group, there were progressive changes in heart rate, cardiac structure, and function. Compared to baseline, heart rate was significantly lower at 12 weeks (Figure 2, panel a), while LVDD/BW and LVSD/BW were significantly increased at 12 weeks (Figure 2, panel b). Similarly, compared to baseline, RWT/BW and fractional shortening (%) were significantly reduced at 12 weeks (Figure 2, panel c and d, respectively).

### Urine and myocardial tissue norepinephrine

**Figure 3:**
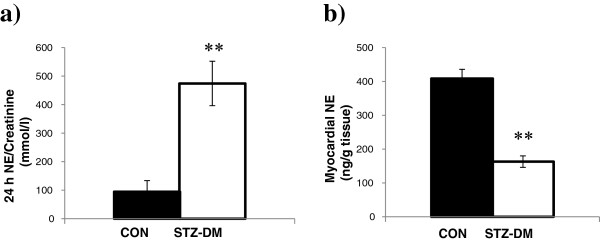
**Urinary and myocardial NE levels in CON (n = 5) and STZ-DM (n = 6). (a)** Urinary NE excretion was measured by enzyme-linked immunoassay. **(b)** Myocardial NE content was measured by high performance liquid chromatography in tandem mass spectrometry. Data are mean ± SEM. *t* test CON vs STZ-DM. **p < 0.001. CON: control; STZ-DM: streptozotocin-induced diabetes mellitus; NE: norepinephrine.

Urinary NE concentrations were greater in STZ-DM animals compared to CON animals (Figure 3, panel a). Myocardial tissue NE concentrations were significantly lower in the STZ-DM animals compared to CON animals (Figure 3, panel b).

### Neuronal markers

**Figure 4:**
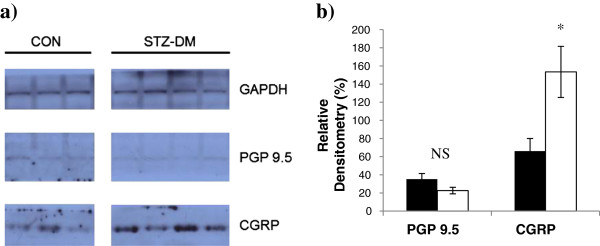
**Myocardial neuronal markers in CON (n = 3) and STZ-DM (n = 4). (a)** Representative PGP 9.5, CGRP, and GAPDH immunoblots from CON and STZ-DM groups. **(b)** Densitometry values for PGP 9.5 and CGRP normalized to GAPDH in CON and STZ-DM groups. *t* test CON vs STZ-DM. *p < 0.05. Data are mean ± SEM. CON: control; STZ-DM: streptozotocin-induced diabetes mellitus; PGP 9.5: protein gene product 9.5; CGRP: calcitonin gene-related peptide; GAPDH: glyceraldehyde-3-phosphate dehydrogenase.

No differences were found in PGP 9.5 levels between groups (Figure 4). In contrast, CGRP levels were greater in the STZ-DM group compared to the CON group (Figure 4). GAPDH expression was similar between groups.

## Discussion

To the best of our knowledge, using a non-insulin-dependent animal model of DM this is the first longitudinal study that addresses the time course and co-occurrence DSPN and DM-induced cardiac structure and function. As early as 4 weeks after the onset of DM, we found evidence of ventricular remodeling and systolic dysfunction, exemplified by decreases in fractional shortening, and DSPN, exemplified as reduced thermal sensation, indicating the development of insensate neuropathy. In addition, both of the latter findings occurred concomitantly with reduced myocardial NE content and altered myocardial CGRP levels, suggesting myocardial neuronal remodeling.

### Development of insensate neuropathy

In DM animal models, manifestations of sensory neuropathy are commonly evaluated by quantitative sensory testing that measures behavioral responses to thermal and mechanical stimuli. These methods are non-invasive and allow for longitudinal evaluation of sensory symptom progression. In insulin-dependent and non-insulin-dependent DM animal models, the development of sensate and insensate neuropathy has been variable, with some investigators reporting one or the other or both. The onset and type of neuropathy appear to be dependent on the animal species and strain [17].

In this study, we found that STZ-DM Fischer 344 rats exhibited reduced sensitivity to both noxious thermal stimuli as well as non-noxious tactile stimuli as early as 4 to 5 weeks after DM induction. This pattern of response is in accordance with several studies that showed impaired sensory perception for tactile and thermal stimuli at 4 weeks [18,19] and 8 weeks [20] post-STZ in mice. Using male Wistar rats and as early as 2 weeks post-STZ, Sugimoto et al. found thermal hypoalgesia in parallel with mechanical hyperalgesia [21]. Others have shown, in male Wistar and female Sprague–Dawley rats, thermal hyperalgesia develops at 4 weeks post-STZ and evolves into thermal hypoalgesia between 8 and 12 weeks [22,23]. In our study, unless pain developed in a transient way in between measurements, there was no evidence of pain at any time point. Discrepancies in the onset and types of neuropathy responses among studies may be attributable to the use of different rodent strains [24,25] with different metabolic characteristics. Specifically, our findings along with others suggest that the Fischer 344 strain may not develop painful neuropathy [26]. Fisher 344 rats, compared to other rodent strains have heightened hypothalamic-pituitary-adrenal axis activity and elevated corticosteroid levels which may render this strain more resistance to inflammation and other metabolic-induced symptoms, such as pain [27]. However, to fully address possible pain development in a Fischer 344 DM model, future studies should include testing with noxious mechanical and chemogenic stimuli.

### Myocardial functional and structural changes

Echocardiography represents a state-of-the-art non-invasive technique for the serial evaluation of functional and structural myocardial changes. Human and animal models of DM cardiomyopathy are characterized by both diastolic and systolic dysfunction. Similar to others [28,29], in the present study, diastolic dysfunction as exemplified by a reduction in the E/A ratio and prolonged IVRT was found at 4 weeks. However, at 4 weeks, we also found systolic dysfunction, exemplified by a decrease in fractional shortening (%), which became progressively lower at both 8 and 12 weeks. With regard to systolic dysfunction, Radovits et al. using a STZ non-insulin dependent DM Sprague-Dawley model, found decreases in ejection fraction and preload recruitable stroke work, a measure of myocardial contractility, at 8 weeks post STZ [30]. In the latter study these contractility parameters were only measured at 8 weeks post STZ injection. Other studies using a similar non-insulin DM model have evaluated cardiac function, however at later time points, such as 2 and half months and 6 months following STZ injection [31,32]. Since our first evaluation of cardiac function was at 4 weeks, it is possible that diastolic and systolic dysfunction could have developed at an earlier time point. Becher et al., using a Sprague-Dawley STZ-DM model (single STZ [70 mg/kg] injection) and using the conductance Millar catheter technique, found significant changes in left ventricular relaxation that occurred at 2 weeks post STZ injection. Impaired relaxation was exemplified by increases in left ventricular diastolic relaxation time (msec) and decreases in left ventricular diastolic relaxation (mm Hg/sec), these changes were also found in conjunction with decreases in cardiac output and decreased left ventricular contractility (mm Hg/sec), indicating reduced systolic function [33]. Changes in cardiac structure, such as cardiac dilation were evident at 4 weeks, exemplified by increased left ventricular end diastolic and systolic dimensions and decreases in relative wall thickness. Similar to others and our findings related to function, we found that myocardial structural changes progressed with the duration of DM [34,35]. We chose to begin our cardiac evaluation at 4 weeks because others [34,36] using a rodent STZ-DM model found DM-induced cardiac changes developed 6–12 weeks after the induction of DM. Findings from our study, suggests that both diastolic and systolic dysfunction develop earlier after the onset of DM.

### Cardiac autonomic regulation and myocardial neuronal markers

Indices of DM cardiac autonomic neuropathy in animal models can be studied by a variety of methods, including analysis of myocardial NE content, cardiac sympathetic and parasympathetic innervation density, and heart rate variability [37]. Twenty-four hour urinary NE excretion is a commonly used biomarker to measure autonomic dysfunction [38]. Changes in plasma and urinary NE levels have been reported in DM animal models [39]. Similar to others, we found increased urinary NE excretion in STZ-DM animals [40]. Interestingly, Tidholm et al. showed that increased NE excretion correlated with reduced myocardial FS (%), increased LVDD and LVSD in dogs with preclinical and clinical DM [41]. Increased urinary NE excretion could be a result of increased NE spill-over due to either increased sympathetic tone or loss of sympathetic nerve terminal function [42].

In DM animal models, results related to myocardial NE levels have been equivocal. Some researchers have reported increases in myocardial NE levels as early as one month following the onset/induction of DM [43] and decreases after 12 months [44]. In the present study, a significant reduction in myocardial NE content was found at 8 weeks, suggesting cardiac sympathetic denervation, impaired release or synthesis. In addition, similar to our study and using a low-dose STZ non-insulin-dependent DM mouse model, Kusmic et al. found increased urinary NE excretion and reduced myocardial NE content at 7 weeks post DM induction [45]. Using imaging of ^123^I-metaiodobenzylguanidine myocardial retention by single-photon emission computed tomography to evaluate the function of sympathetic neuronal endings, Kusmic et al. found accelerated tracer washout, indicating reduced NE uptake, and they suggested this might be due to decreased NE transporter expression [45]. In the present study, we also found a significant reduction in heart rate. Collectively, our data support altered myocardial autonomic control/dysfunction. Future studies should measure myocardial and urinary NE levels before and after 8 weeks as well as other markers of cardiac autonomic dysfunction such as heart rate variability.

Deficits in cardiac sensory afferents, another aspect of DM cardiac pathology that may reflect neuropathy, have been linked to changes in sensory neuronal markers such as CGRP [46]. Using STZ mice, Ieda et al. found a decrease in CGRP immunoreactivity and CGRP-specific denervation in DM hearts at 16 weeks but not at 8 weeks of DM [46]. In female Wistar rats, Chottova et al. found increases in CGRP content in DM hearts at 4, 8, and 16 weeks after STZ, as a result of accumulation at the dystrophic terminals [47]. Together, these observations may reflect early dysfunction at the nerve terminal, with impaired release of neurotransmitters (elevated CGRP) and later events associated with cardiac denervation (decreased CGRP). In the present study, we observed elevated myocardial CGRP protein levels at 8 weeks, suggesting early terminal dysfunction with CGRP accumulation and impaired release. On the other hand, cardiac neuronal remodeling affecting sympathetic and parasympathetic control of cardiac function have been studied using staining for acetylcholinesterase [48], molecular imaging [49] and pan-neuronal markers such as PGP9.5 [50]. In cardiac tissue, PGP9.5 is expressed by parasympathetic, sympathetic and sensory nerves and intracardiac neurons. Our results showed no difference in PGP9.5 immunoreactivity in whole cardiac tissue suggesting that at least PGP9.5 positive neurons are not widely affected. Future studies should test for local staining of these markers to better evaluate regional remodeling of neuronal subpopulations.

### Limitations

We have qualified our STZ model, as non-insulin dependent, but would not refer to as a type 2 model, since we believe it more closely resembles type 1 diabetes. Therefore, our results cannot be applied to other types of diabetes. We measured behavioral responses to noxious thermal and non-noxious tactile stimuli and did not evaluate for chemogenic pain or vibration changes. As noted above these latter measurements should be incorporated into future studies. Finally, for our cardiac neuronal studies our sample size was small, therefore these results are qualified as ‘preliminary’ and interpreted with caution.

## Conclusions

In summary, in a non-insulin-dependent STZ-DM rodent model and as early as 4 weeks after the onset of DM, we found evidence of the co-occurrence of ventricular remodeling, systolic dysfunction, and DSPN. Reduced myocardial NE content, reduced heart rate, and altered myocardial levels of biomarkers of sensory innervation may indicate the presence of cardiovascular autonomic neuropathy. Understanding the onset and temporal relationship of common complications in animal models frequently used in pre-clinical studies, may lead to a better understanding of the pathophysiology underlying these complications. These studies also suggest the need to screen for cardiovascular dysfunction in DM patients presenting with insensate neuropathy.

## Abbreviations

BW: Body weight; CGRP: Calcitonin gene-related peptide; CON: Control; DM: Diabetes mellitus; DSPN: Diabetic symmetrical polyneuropathy; E/A: Ratio e wave, a wave; GAPDH: Glyceraldehyde-3-phosphate dehydrogenase; HR: Heart rate; IVRT: Isovolumic relaxation time; IVSD: Interventricular Septum in diastole; IVSS: Interventricular septum in systole; LVDD: Left ventricular diameter in diastole; LVSD: Left ventricular diameter in systole; NE: Norepinephrine; PGP 9.5: Protein gene product 9.5; RWT: Relative wall thickness; STZ: Streptozotocin; STZ-DM: Streptozotocin-induced diabetes mellitus.

## Competing interests

The authors declare that they have no competing interests.

## Authors’ contributions

MNM, STB, and MRP contributed to the conception and design of experiments. All authors contributed to the acquisition, analysis and interpretation of data. MNM and MRP drafted and wrote the article. All authors critically revised, edited, and approved the final version of the submitted manuscript.

## Supplementary Material

Additional file 1 **Chromatogram comparing homogenization buffer with and without sodium metabisulfite as antioxidant.** Purple trace: 5 mM NE with 0.1% Na_2_ (SO_3_), Blue trace: 5 mM NE without 0.1% Na_2_ (SO_3_), Red trace: water blank. Click here for file
