# Supplementary figures and images for: The co-occurrence of myocardial dysfunction and peripheral insensate neuropathy in a streptozotocin-induced rat model of diabetes
Source: Cardiovasc Diabetol. 2014 Jan 11;13:11. doi: 10.1186/1475-2840-13-11 (PMC3893387; doi:10.1186/1475-2840-13-11)

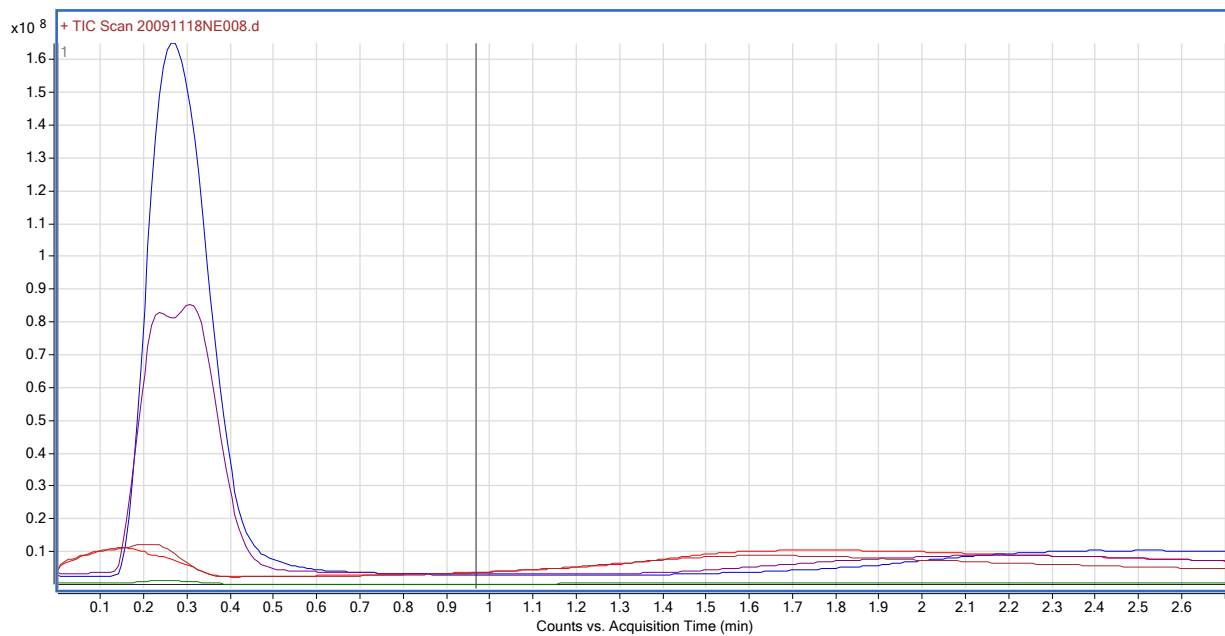

Supplement: Additional file 1 — Chromatogram comparing homogenization buffer with and without sodium metabisulfite as antioxidant. Purple trace: 5 mM NE with 0.1% Na2 (SO3), Blue trace: 5 mM NE without 0.1% Na2 (SO3), Red trace: water blank. [file 1475-2840-13-11-S1.pdf]
